# How Musical Training Shapes the Adult Brain: Predispositions and Neuroplasticity

**DOI:** 10.3389/fnins.2021.630829

**Published:** 2021-03-10

**Authors:** Alicja M. Olszewska, Maciej Gaca, Aleksandra M. Herman, Katarzyna Jednoróg, Artur Marchewka

**Affiliations:** ^1^Laboratory of Brain Imaging, Nencki Institute of Experimental Biology of the Polish Academy of Sciences, Warsaw, Poland; ^2^Laboratory of Language Neurobiology, Nencki Institute of Experimental Biology of the Polish Academy of Sciences, Warsaw, Poland

**Keywords:** neuroplasticity, neuromusicology, neuroimaging, reorganization, skill learning, music, training, predispositions

## Abstract

Learning to play a musical instrument is a complex task that integrates multiple sensory modalities and higher-order cognitive functions. Therefore, musical training is considered a useful framework for the research on training-induced neuroplasticity. However, the classical nature-or-nurture question remains, whether the differences observed between musicians and non-musicians are due to predispositions or result from the training itself. Here we present a review of recent publications with strong focus on experimental designs to better understand both brain reorganization and the neuronal markers of predispositions when learning to play a musical instrument. Cross-sectional studies identified structural and functional differences between the brains of musicians and non-musicians, especially in regions related to motor control and auditory processing. A few longitudinal studies showed functional changes related to training while listening to and producing music, in the motor network and its connectivity with the auditory system, in line with the outcomes of cross-sectional studies. Parallel changes within the motor system and between the motor and auditory systems were revealed for structural connectivity. In addition, potential predictors of musical learning success were found including increased brain activation in the auditory and motor systems during listening, the microstructure of the arcuate fasciculus, and the functional connectivity between the auditory and the motor systems. We show that “the musical brain” is a product of both the natural human neurodiversity and the training practice.

## Introduction: What is Neuroplasticity? Why is it so Important to Study it?

The constantly changing environment, the drive for new knowledge and skills, all require behavioral flexibility. The brain, as the source of behavior, adapts its architecture and functions to perform new tasks through processes broadly defined as neuroplasticity. These processes include, among others, dynamic reconfiguration of neural connections, cell shape, size, myelination, synaptic strength and neurogenesis, the last one limited to the olfactory bulb and the hippocampus in adults (Tardif et al., 2016). In human neuroimaging studies, it is possible to indirectly measure macroscopic effects of the neuroplastic biological dynamics via functional and structural modalities (for the overview of the relationship between macroscopic measures and the underlying biology, see Tardif et al., 2016). Although usually measured separately, functional and structural neuroplasticity reflect various aspects of the same neuroplastic processes and are thus inherently intertwined in a complex manner.

We currently understand that the human brain is not shaped exclusively during critical periods of development. Neuroplastic changes occur in response to internal and external stimuli throughout the entire lifetime (Draganski and May, 2008). From a social perspective, neuroplasticity processes underlie such phenomena as education, neurological rehabilitation, or healthy aging.

## Musical Training as a Framework for Studying Brain Plasticity

Generally, in studies on neuroplasticity, two questions arise: what are the structural and functional changes related to a particular behavioral need, and how do they occur over time. To effectively answer these questions, we first need to elicit a novel behavior. There is a wide spectrum of learning protocols which were employed so far to understand neuroplasticity. Simple ones engage only a single sensory modality, like auditory (Zatorre et al., 2012) or tactile (Hodzic, 2004). More complex ones utilize sensorimotor associations and higher-order cognitive functions tasks, like the acquisition of foreign languages or tactile reading (Li et al., 2014; Siuda-Krzywicka et al., 2016). The complexity of music performance requires a unique and multi-system involvement from the human brain (Münte et al., 2002; Herholz and Zatorre, 2012; Schlaug, 2015). Playing a musical instrument requires sensorimotor adaptations, as with the use of any tool, and more: a mapping of specific movements to the auditorily perceived outcomes, which follow a set of more or less intuitively understood rules of musical harmony, esthetics and pleasure. It comprises both feed-forward and feedback interactions between the integrated multisensory input (tactile, proprioceptive, auditory, and visual) with motor output, as well as higher-order cognitive functions such as memory, attention, emotion, and the processing of musical syntax (Zatorre et al., 2007; Brown et al., 2015). Additionally, as rewarding stimuli are learned better than non-rewarding ones (Schultz, 2000), it is likely that the highly rewarding nature of musical performance promotes learning and drives brain plasticity (Penhune, 2019). Therefore, learning to play a musical instrument provides a useful framework to study multimodal brain plasticity.

Secondly, the changes in brain structure and function have to be sampled frequently enough to capture the dynamics of the neuroplastic processes. Brain volume changes do not relate to practice in a monotonically increasing way (Lövdén et al., 2013; Wenger et al., 2017). Yet, we observe continuous behavioral improvement and the extent of behavioral and plastic changes correlate with training duration. The proposed model of neuroplasticity includes a period of initial growth, after which comes a renormalization phase, when the efficiency of brain circuits increases while cortical volume does not (Wenger et al., 2017). From a functional perspective, plastic changes can be reflected in increased functional activation of a brain area related to a function, its expansion on neighboring areas, or an involvement of novel, often distant, areas. Interestingly, cortical map plasticity may also follow a comparable pattern of expansion followed by retraction to pre-training levels during learning as seen in structural changes (for review see Wenger et al., 2017). Therefore, the functional (and structural) expansion temporarily increases the available pool of circuits to be used “exploratively” until the most efficient circuit to perform the task is determined. As learning continues, the selected circuitry is further stabilized through practice, the performance increasingly relies on that circuit and thus the cortical map renormalizes (Wenger et al., 2017).

Two experimental approaches are typically employed in cognitive neuroscience to understand brain reorganization following training, namely the cross-sectional and the longitudinal design. Comparing musically naive and proficient individuals in cross-sectional studies can provide important insights into the neuroplasticity of the human brain (Münte et al., 2002). Musicians practice musical performance regularly for most of their lives, often starting in early childhood and practising for many years. Juxtaposing musicians and non-musicians can show changes associated with very long training. However, while it might be tempting, the causal relationship between musical training and the observed differences cannot be inferred from correlational studies (Schellenberg, 2019). The cross-sectional study design does not reveal the time course of the plastic changes nor does it correct for any possible predispositions. To infer causality, a theoretical model needs to be constructed and validated against properly designed longitudinal studies. Longitudinal studies can account for the interindividual variability pre-training, but are costly, with costs increasing with the duration of the experiments.

Finally, advances in non-invasive neuroimaging methods gave scientists specific tools to non-invasively study brain plasticity in living humans. Structural and functional neuroimaging techniques were used to compare brain anatomy and function between groups of musicians and non-musicians, and, more recently, to study the plastic changes related to musical training in longitudinal studies.

This review aims to present the newest evidence for experience-related neuroplasticity in the context of musical training in adults, concentrating on neuroimaging and with an emphasis on longitudinal studies. Since the scope of this review is limited, and the focus is on musical training as a model for studying brain plasticity in neurotypical adults, studies of complex developmental and aging-related changes are not discussed. We particularly focus on experimental designs in order to better understand both brain reorganization and the neuronal markers of predispositions when learning to play a musical instrument. Since we include studies which use a multitude of functional as well as structural neuroimaging techniques, we also provided a brief overview of such methods highlighting the advantages and disadvantages of each method for neuroplasticity research (Table 1).

**TABLE 1:** An overview of neuroimaging methods used to study neuroplasticity in humans.

Modality	Information obtained	Advantages for neuroplasticity research	Limitations in neuroplasticity research
**Structural**			
Magnetic Resonance Imaging (MRI)	• Morphological characteristics assessed with voxel-based morphometry (VBM; to assess gray volume/density) or indexes of cortical thickness. • Plasticity specific: Information about alteration in the brain’s macroscopic structure, with various plausible underlying biological mechanisms (synaptogenesis, neurogenesis, gliogenesis, angliogenesis).	• Plasticity specific: Information about alteration in the brain’s macroscopic structure, with various plausible underlying biological mechanisms (synaptogenesis, neurogenesis, gliogenesis, angliogenesis). • Does not require performance of the task in the scanner.	• In cross-sectional studies, structural methods may not only detect differences that are specific to learning the skill of interest but also more general group differences (professional musicians who devoted years to musical training may be more persistent that novices, which may be reflected in brain structure). For further limitation of structural neuroimaging techniques in neuroimaging research see Thomas and Baker (2013).
Diffusion-based MRI (Diffusion Tensor Imaging; DTI)	• Information about the white-matter integrity, microstructure, and white matter connections based on the directional asymmetry of water diffusion. Fractional anisotropy (FA) is a global estimate of the linearity of diffusion, while mean diffusivity (MD) quantifies the amount of diffusion in each voxel. Higher FA and lower MD denote higher levels of organization of the white matter structure.	• Quantification of white matter integrity as well as delineation of white matter pathways connecting different regions of the brain associated with expertise or changes during learning. • Does not require performance of the task in the scanner.	
	• Plasticity specific: Information about regional reorganization of myelinating tracts, with various plausible underlying biological mechanisms (myelination, synaptogenesis, neurogenesis, gliogenesis).		
**Functional**			
Task-related Functional MRI (fMRI)	• Detection of changes in the blood oxygenation level-dependent (BOLD) signal, which is affected by changes in neural activation in a specific brain region and the underlying physiology. • Localization of brain activation associated with performing a cognitive task and/or behavior.	• Detection of changes in brain activation while individuals perform a task of interest (e.g., playing an instrument).	• Task-related methods may not always be suitable for cross-sectional studies (as novices will not be able to perform the task) or for tasks that are impossible to perform in the scanner environment. • In longitudinal research, may detect changes in activation not specific to learning a new skill, but instead changes in perceived task-difficulty or awareness of task sequential structure (for further discussion see Poldrack, 2000).
			• In isolation, task-related activations/deactivations are ambiguous and may reflect compensatory activations, task performance automatization or underlying changes in vascular or metabolic environment.
Task-related Electroencephalogram (EEG) & Evoked Related Potentials (ERP)	• Direct recording of underlying electrical brain activation associated with a cognitive task and/or behavior.		• Requires a carefully selected control condition.
Task-related Magnetoencephalography (MEG)	• Direct recording of brain activation, assessing brain magnetic fields.		
Functional Connectivity (FC)	• Functional interactions (neural synchronization) between different brain regions.	• Does not require performance of a task in the scanner. • Possible to compare experts and novices, or track connectivity longitudinally.	• In cross-sectional studies, may not only detect differences that are specific to learning the skill of interest but also more general group differences (for example, professional musicians who devoted years to musical training may be more persistent that novices, which may be reflected in FC).

## Insights From Musical Training

### Cross-Sectional Studies

Anatomical studies postulated that musicians’ compared to non-musicians’ brains developed differently in several brain structures, in particular in temporal and frontal areas (Gaser and Schlaug, 2003; Schneider et al., 2005; Bermudez et al., 2009; Elmer et al., 2013; Groussard et al., 2014; James et al., 2014; Sato et al., 2015; Karpati et al., 2017). Since the primary auditory cortex constitutes an integral part of the temporal lobe, increased gray matter volume or cortical thickness in this area can be linked to better sound perception. In the frontal lobe, musicians have larger gray matter volume most notably in areas related to executive functions (i.e., bilateral inferior frontal gyri, middle and superior frontal gyri), such as maintenance, monitoring and retrieval of musical information, as well as the processing of musical structures (Bermudez et al., 2009; Groussard et al., 2014; James et al., 2014; Sato et al., 2015). Compared to naïve controls, professional musicians have also higher gray matter volume in the hippocampus, which is widely connected to memory-related processes (Gaser and Schlaug, 2003; Schneider et al., 2005; Bermudez et al., 2009; Elmer et al., 2013; Groussard et al., 2014; James et al., 2014; Sato et al., 2015; Karpati et al., 2017). In addition, musicians have larger gray matter volume in lingual gyrus, implicated in musical score reading and visuospatial transformations, allowing musicians to read notes into the instrument (Bermudez et al., 2009; Sato et al., 2015; Vaquero et al., 2016). They also have greater cortical thickness in the primary somatosensory cortex, which can be linked to physically having contact with a musical instrument (Bermudez et al., 2009; Elmer et al., 2013; Karpati et al., 2017). These findings suggest that learning to play musical instruments strongly influences the organization of gray matter in multiple brain networks related to sensory processing (particularly auditory, vision, and somatosensory regions) and higher-order cognitive function.

Studies using DTI present a pattern that corresponds well to the aforementioned gray matter volume results. They show that musicians have more developed white-matter connections between motor cortex and the spinal cord (Giacosa et al., 2016), as well as occipital lobe with anterior temporal regions (Schmithorst and Wilke, 2002; Giacosa et al., 2016). Arcuate fasciculus, the fiber tract connecting motor and auditory areas, shows greater white matter organization (higher fractional anisotropy) in musicians than non-musicians (Halwani et al., 2011). Additionally, expert players have higher fractional anisotropy in the corpus callosum - the most abundant white matter tract in the brain connecting brain hemispheres (Schmithorst and Wilke, 2002; Steele et al., 2013; Schlaffke et al., 2020). Similarly, greater fractional anisotropy was observed in cerebellum and striatum, which are essential in learning repetitive and automated finger movements (Schmithorst and Wilke, 2002; Abdul-Kareem et al., 2011), and in the tract connecting the right middle temporal gyrus and the pars opercularis, thought to mediate musical syntax processing (Oechslin et al., 2018). Together, the results from cross-sectional studies suggest that musical training is associated with distinct changes in white matter architecture, particularly in regions linked to fine motor control and sensory processing.

Functional magnetic resonance imaging (fMRI) studies employing passive listening tasks revealed that musicians, compared to non-musicians, had extended activations in the temporal areas linked to improved auditory processes, including the extraction, processing and comparison of pitch information (Ohnishi, 2001; Seung et al., 2005; Bangert et al., 2006; Limb et al., 2006; Habermeyer et al., 2009; Bianchi et al., 2017). A comparable outcome was observed in the frontal lobe, in the primary and supplementary motor areas, related to motor preparation and execution, and in areas related to language processing, such as Broca’s area (Bangert et al., 2006; Limb et al., 2006; Habermeyer et al., 2009; Bianchi et al., 2017). Moreover, some studies presented increased activations in parietal areas - primarily in the supramarginal gyrus, which can be connected to syntax processing and selective attention in musical stimuli (Seung et al., 2005; Limb et al., 2006; Oechslin et al., 2013). Importantly, in the aforementioned studies, the differential brain activation pattern was correlated with behavioral measures of musical aptitude. Thus, these findings suggest that better musical abilities in musicians are reflected in training-induced neuroplastic changes, particularly increased activation of brain areas associated with auditory processing, motor responses, as well as attention while listening to the music.

In studies employing EEG and MEG during passive music listening tasks, musicians show a higher amplitude of brain-generated electrical or magnetic potential (event-related potential) in the frontal cortex (Rigoulot et al., 2015) compared to non-musicians. Furthermore, in an experiment that included musical pieces with incongruent endings, musicians had a higher response in temporal-limbic areas, associated with memory and processing of emotions (James et al., 2008). Additionally, musicians presented higher mismatch negativity responses in auditory areas (Herholz et al., 2011; Kuchenbuch et al., 2014). This may imply that musicians have a more developed pre-attentive auditory processing than non-musicians.

**FIGURE 1:**
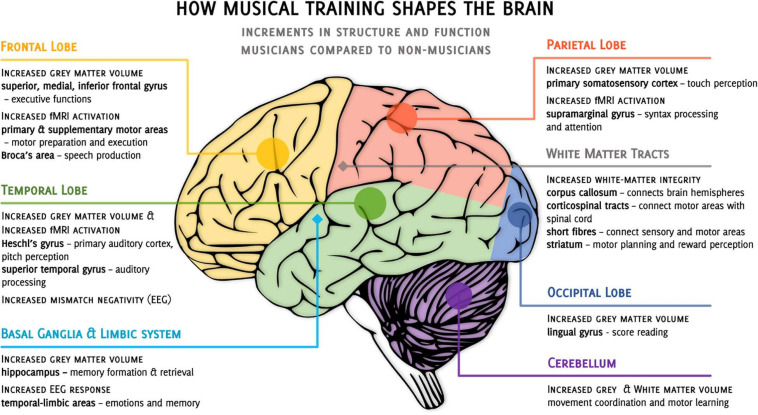
Overview of cross-sectional studies on the incremental differences observed when comparing musicians to non-musicians. •, cortical structures, ◆, subcortical structures, fMRI, functional magnetic resonance imaging, EEG, electroencephalography.

In summary, cross-sectional studies in different neuroimaging modalities show one fairly consistent image - musicians differ from naive participants in both structure and functional response of the brain (Figure 1). However, it is not clear to what extent the differences between musically naive and proficient individuals are the result of musical training itself. Based on cross-sectional studies only, it is not possible to exclude that these differences are, at least partially, a consequence of some natural predispositions; a typical nature-or-nurture question (Zatorre, 2013; Zuk and Gaab, 2018). In favor of training-related plasticity speaks research showing that the age of the musical training onset (Schlaug et al., 1995; Amunts et al., 1997; Steele et al., 2013), a longer duration of training (Elbert et al., 1995; Groussard et al., 2014), or greater training intensity (Gaser and Schlaug, 2003; Bengtsson et al., 2005; Schneider et al., 2005) correlate with the magnitude of white and gray matter changes. However, recent estimates indicate that differences in training intensity can only explain from 21 to 36% of the variance in expert performance (Macnamara et al., 2014; Platz et al., 2014), with longer practice not guaranteeing musical expertise (Mosing et al., 2014).

Research on monozygotic twins could shed some light on the training-induced neuroplasticity versus natural predispositions hypotheses of neural differences between musically trained and naïve individuals. We are aware of only one neuroimaging experiment conducted on monozygotic twins discordant for musical training, with the siblings’ within-pair differences of total music practice ranged between 1768 and 9516 h (M = 4220 ± 2730) (de Manzano and Ullén, 2018). None of the study participants was a professional musician. This study revealed increased cortical thickness in the left cerebral auditory-motor network, greater gray matter volume in cerebellar lobules I-IV + V, and higher fractional anisotropy of the key white-matter tracts that enable auditory-motor integration and motor execution (i.e., arcuate fasciculus, inferior longitudinal fasciculus, uncinate fasciculus, and corticospinal tracts) and the corpus callosum in the musical siblings compared to their twins. As many factors which might predispose someone to musical proficiency are shared between twins, such as genes and early environment, the differences between musically proficient twins and their non-musician siblings may be attributed to musical training.

### Limitations of Cross-Sectional Studies and Suggestions for Future Research

Cross-sectional studies are observational studies that analyze data at one specific point in time. As there is no retrospective or prospective follow-up, cross-sectional studies are inexpensive and easy to conduct, making them an excellent tool to observe certain effects, without long-term, expensive research (Wang and Cheng, 2020). Despite these advantages, cross-sectional studies have a number of important limitations. While differences between musicians and non-musicians can be observed this way, these differences cannot be attributed directly to musical training. Thus, it is impossible to establish a direct causal link between musical training and differences in the brain’s structure or function. Cross-sectional studies provide information about the participants’ degree of musical expertise at the time of the study, but they do not offer insight into the inherent difference in factors predisposing to musical ability. In other words, there is no information why some of the participants decided to pursue a musical path, while others either have not or have never even considered it in the first place. In effect, we cannot exclude the possibility that musicians and non-musicians in such studies are samples of two different populations and the results reflect pre-existing differences predisposing to musical ability rather than the effect of musical training *per se*.

A substantial amount of cross-sectional studies presented in this paper had a relatively small sample of 20 or fewer participants per group (Elmer et al., 2013; Groussard et al., 2014; Karpati et al., 2017). Such a sample could be considered valid only if the actual difference between musicians and non-musicians constitute a large effect size. Such studies might be statistically underpowered and thus susceptible to inflated effect size (Button et al., 2013). Therefore, it is crucial to consider their results with a degree of caution. Some of the studies mentioned did not use (or did not mention the use of) any multiple comparisons correction (Elmer et al., 2013; Groussard et al., 2014; James et al., 2014).

To increase the reliability of the findings and in particular the statistical power, future cross-sectional studies should substantially increase sample sizes and employ a reproducible and statistically valid method of analysis with multiple-comparison correction.

### Longitudinal Studies

The longitudinal approach gives a unique opportunity to track temporal dynamics of brain changes during learning. Moreover, interindividual differences and predispositions can be measured before the training commences. Therefore, the outcome of training can be attributed to neuroplasticity, not the pre-existing factors. Up to now, only a few studies were conducted using musical training in a longitudinal design. However, those experiments differ greatly in their methodologies, such as the number of measurements (time points), the interval between them, the presence of control groups or control conditions, and outcome measures (see Table 1 for summary). Although most of the research concentrated on piano training (Chen et al., 2012; Herholz et al., 2016; Brown and Penhune, 2018; Li et al., 2018, 2019; Tavor et al., 2020), one study was conducted using cello (Wollman et al., 2018) and one using drums training (Amad et al., 2016). One common aspect was the use of “listening to trained melodies” as one of the experimental tasks while the activation of participants’ brain was being examined with fMRI (Chen et al., 2012; Herholz et al., 2016; Brown and Penhune, 2018; Wollman et al., 2018), MEG (Lappe et al., 2008; Pantev et al., 2009) or EEG (Bangert and Altenmüller, 2003) methods. Other researchers investigated training-related plasticity of the resting-state functional connectivity (Li et al., 2018; Wollman et al., 2018), white matter connectivity (Li et al., 2018), white matter diffusivity (Tavor et al., 2020), or network flexibility (Li et al., 2019). In some experiments (Chen et al., 2012; Brown and Penhune, 2018; Vaquero et al., 2018; Tavor et al., 2020), short-term plasticity was studied through training duration of several minutes to hours. Others (Amad et al., 2016; Li et al., 2018, 2019; Wollman et al., 2018) investigated more long-term effects, observed after weeks or months of training.

#### MRI Neuroimaging Studies

Two fMRI studies, which employed a short-duration piano training inside an MRI scanner, presented somewhat conflicting results (Chen et al., 2012; Brown and Penhune, 2018). In the first one (Chen et al., 2012) the activation of the left dorsal and ventral premotor cortices decreased as the training progressed, as did the activation of the bilateral superior temporal gyrus during a listening task in late compared to early training, while the activation of right retrosplenial cortex, left orbitofrontal cortex and frontal pole showed increased activation in the late vs early phase of training. On the other hand, the study by Brown and Penhune (2018), reported an increased activation of the occipital, temporal, premotor cortex and the cerebellum for late compared to early training. These discrepancies might be attributed to substantial differences in training and testing protocols and contrasts used in the two studies. Nevertheless, the involvement of the premotor regions and the superior temporal gyrus in learning a musical piece is not surprising, as they are parts of the dorsal auditory streams, where sound and movement are integrated into the context of music and speech (Zatorre et al., 2007). The dorsal auditory streams are responsible for sensorimotor integration and spatial processing (Rauschecker and Tian, 2000), which is necessary for the auditory-motor transformations that allow humans to play music. Chen et al. (2012) argued that the reduced activation in brain regions belonging to the dorsal auditory stream suggests increased efficiency in neural processing of a learned stimulus. However, Brown and Penhune (2018) interpret an increase in the activation of the premotor areas across learning trials as a probable reflection of the auditory-motor associations. In other studies, decreased activation of the superior temporal gyrus was associated with perceptual pitch training (Jäncke et al., 2001) and with micromelody discrimination training (Zatorre et al., 2012). Additionally, in the experiment of Brown and Penhune (2018), other notable changes associated with training included decreased activation during training tasks in the parietal cortex, which might facilitate the translation of pitch patterns into movement (Gnadt and Andersen, 1988; Mattingley et al., 1998), and the anterior cingulate cortex, involved in error detection and correction during learning. Interestingly, recent evidence suggests the anterior cingulate cortex activation during listening to dissonant compared to consonant music stimuli (Bravo et al., 2020) and its involvement in singing and playing the cello by professional cellists (Segado et al., 2018).

Lately, Tavor et al. (2020) used DTI approach to study short-term changes in neuroplasticity: immediately before and after each of the two piano training sessions. After the first training session of 45 min, a significant reduction of 2–3% in mean diffusivity (suggesting an increase in white matter organization) was found in the left premotor cortex, the left middle temporal gyrus, and bilaterally in the superior part of the cerebellum. Additional mean diffusivity reduction in the lingual gyrus was found after the second training session of 40 min. These timescales are consistent with astrocyte remodeling (Blumenfeld-Katzir et al., 2011; Sagi et al., 2012; Assaf, 2018), which might be indicative of long-term potentiation, a primary cellular mechanism of neuroplasticity. Previously, structural differences within the white matter of the cerebellum, a known structure facilitating motor learning and movement coordination, were identified in cross-sectional DTI studies comparing musicians and non-musicians (Schmithorst and Wilke, 2002; Abdul-Kareem et al., 2011). Moreover, a larger volume of the lingual gyrus, involved in musical score reading, was found in musicians compared to non-musicians (Bermudez et al., 2009; Sato et al., 2015; Vaquero et al., 2016). As the experiment by Tavor et al. (2020) involved no actual reading of the musical score, and the participants learned to play by viewing and hearing a sequence played on a virtual keyboard, it is more probable that the engagement of the lingual gyrus was related to the processing of complex visual stimuli. However, the authors argue that the plasticity in the lingual gyrus might reflect its direct involvement in the motor or auditory aspects of the tasks (Janata, 2002; Müller et al., 2002; Schmithorst and Holland, 2003; Parsons et al., 2005; Bengtsson et al., 2009). Crucially, the results by Tavor et al. highlight that the brain is an extremely dynamic structure with tissue plasticity occurring even over short timescales.

In recent years, several experiments measured changes on a longer timescale of weeks to months. A study on drums training (Amad et al., 2016), investigated changes in resting-state functional connectivity before and after 8 weeks of learning. Multivariate pattern analysis of the whole-brain connectome identified that drum training was associated with increased functional connectivity between the posterior part of bilateral superior temporal gyrus and the rest of the brain. The bilateral superior temporal gyrus was previously recognized in fMRI studies as involved in action-representation of sounds in musical tasks such as drumming (Tsai et al., 2012) and learning to play the piano in the abovementioned work (Chen et al., 2012; Brown and Penhune, 2018). Follow-up seed-to-voxel analysis revealed increased functional connectivity between the posterior and middle parts of the superior temporal gyrus (seed) and the motor and premotor brain regions and the right parietal lobe. The activation of premotor cortices is coupled with that of the auditory cortex in the music context, showing co-activation during listening to a melody previously learned on a keyboard, but not other melodies (D’Ausilio et al., 2006; Lahav et al., 2007). This co-activation was also observed in professional pianists who listened to musical pieces they knew how to play, and played them without auditory feedback (Zatorre et al., 2007). The coupling between the auditory and the (pre)motor brain areas seems to be involved in music production. The increase in functional connectivity between the posterior superior temporal gyri seed and the right parietal lobe cluster, which belongs to the dorsal attention network, was correlated with the improvement in drum performance. The authors postulate this might be related to the improvement of the integration of sensory and motor functions due to drum training. Moreover, the superior parietal lobule is involved in motor learning, sensorimotor and somatosensory integration, among other sensory and cognitive processes (Zatorre et al., 2007; Wang et al., 2015). It was suggested to play a role in the coordination of spatial and timing components of musical performance (Langheim, 2002) and somatic perception of bimanual object interaction (Naito et al., 2008).

Additionally, functional connectivity decreased between the superior temporal gyrus seed and the cerebellum, what might indicate an increase in efficiency of the cerebellar regions in response to motor learning (Petrini et al., 2011; Vahdat et al., 2011) during drum training.

In another fMRI study (Herholz et al., 2016), participants underwent six weeks of piano training. The activation of the left dorsal premotor cortex increased after training while listening to a familiar melody compared to a random tone sequence. This replicates previous results on the activation of premotor regions when listening to musical pieces one can perform (D’Ausilio et al., 2006; Lahav et al., 2007). Moreover, imagining and listening to trained compared to untrained melodies elicited increased activation in the premotor and dorsolateral prefrontal cortices, posterior parietal cortex bilaterally, and the cerebellum after training. The dorsolateral prefrontal cortex, together with parietal regions, is thought to store representations of learned sequences (Doyon and Benali, 2005; Penhune and Steele, 2012). Increased activation in the parietal lobe stands in opposition to the findings of Brown and Penhune (2018), however, it is hard to interpret this difference given the different paradigms and timescales of these experiments.

More recently, Wollman et al. (2018) investigated brain activation and functional connectivity changes related to 4 weeks of cello training. After the training, increased activation in the supplementary motor areas (pre-SMA & SMA), the right dorsal premotor cortex and the left posterior parietal cortex was observed during a listening task. This functional reorganization, in general, follows the patterns discussed previously, namely the altered response of the premotor and parietal regions of the dorsal stream, occurring after training while listening to trained melodies. Additionally, supplementary motor areas were involved as well, which also belong to the dorsal auditory-to-motor cortical pathway. Similar structures were activated while playing in the scanner as in the listening task, providing further evidence for the close coupling between those areas for musical tasks. Importantly, cello playing and singing by expert cellists evokes overlapping activations in the dorsal premotor cortices, the supplementary and primary motor areas, and the intraparietal sulcus (Segado et al., 2018). Further, resting-state connectivity between the superior parietal lobe and bilateral auditory cortex as well as between the superior parietal lobe and the SMA is positively correlated with performance on musical tasks (Segado et al., 2018). These findings corroborate the outcomes of the drum training experiment (Amad et al., 2016) showing the increased co-activation between premotor and parietal regions, even though widely different instruments were used. In contrast to the studies on piano-training, the activation changes in the frontoparietal network occurred for non-trained melodies but were not seen in the task-related functional connectivity. This difference might stem from the fact that piano is an equal-tempered instrument, and cello is a non-tempered one, where a single pitch can be played in multiple ways. Although no differences were found in brain responses while playing the cello between the first and the fourth week of training, the authors argue that such changes could have occurred during the very first week of practice.

In the longest reported study so far, participants were scanned with resting-state fMRI (rs-fMRI) and DTI before and after a 24-week intensive piano training (Li et al., 2018, 2019). In case of the rs-fMRI, a significant interaction with a control group over time was found in areas of the bilateral postcentral, left superior and right inferior parietal gyrus as well as right precentral gyrus, and right superior frontal gyrus. Additionally, musical training was associated with increased functional connectivity between the right postcentral and the right precentral gyri, and between the auditory and the motor networks. Importantly, functional connectivity within the sensorimotor network and structural connectivity of the auditory-motor network were found to be positively correlated with practice time. These outcomes corroborate previous findings on the changes in the auditory-motor-parietal network related to musical training. Moreover, fractional anisotropy of the corticospinal tract, the superior longitudinal fasciculus, and the corpus callosum after training was increased in the training group compared to controls. Increased fractional anisotropy of the corticospinal tract and the corpus callosum in musicians compared to non-musicians was identified in cross-sectional studies (Schmithorst and Wilke, 2002; Steele et al., 2013; Giacosa et al., 2016; Schlaffke et al., 2020). The dynamics of the resting-state functional connectivity was also analyzed using graph approach, where network flexibility was used as a measure of changes in the local properties of individual elements of a neural network (Li et al., 2019). This analysis yielded similar outcomes. This was the only study on musical training so far to include a follow-up period, which lasted for 3 months after the training was completed. The trainees’ increased connectivity and flexibility related to the 24 weeks of piano training returned to baseline at 12 weeks after the cessation of training (Li et al., 2019, 2018) suggesting that any training-induced alterations are quickly lost if the skills are not used. Although there are no other studies on musical training which included a follow-up period, recent studies on tactile reading (Matuszewski et al., 2021), second language acquisition (Kuper et al., 2021) and sign language learning (Banaszkiewicz et al., 2021) also show a decrease in myelination, but not necessarily in gray matter volume or brain activation, after the cessation of trainings.

#### MEG/EEG Studies

Few MEG studies investigated the influence of musical training on brain activation. In each of them, participants were divided into groups for auditory-sensorimotor training (playing the piano, experimental group) or auditory training (listening and judging the correctness of a heard performance, control group). In one of these studies, the training consisted of eight sessions of 25 min over two weeks. MEG recordings were performed before and after training (Lappe et al., 2008; Pantev et al., 2009). A greater increase in mismatch negativity amplitude in the auditory cortex was found after training in the piano training group compared to the control group indicating greater enhancement of auditory cortex’s response to musical stimuli. Similarly, in an experiment by Paraskevopoulos and colleagues (Paraskevopoulos et al., 2012), participants were divided into an auditory-visual-sensorimotor (experimental) and auditory-visual only (control) training groups. Trainees took part in eight sessions spread over 10 days, lasting 58 min each. The interaction with the control group over time showed increased amplitude of the mismatch negativity in the Brodmann’s area 22 (superior temporal gyrus) during an audio-visual task, with a right-hemisphere dominance. Those outcomes confirm the results of previous studies of increased engagement of the auditory association cortex to musical stimuli after musical training (Lappe et al., 2008; Pantev et al., 2009).

To our knowledge, only one longitudinal EEG study on musical training was conducted so far (Bangert and Altenmüller, 2003). In this study with 10 piano training sessions over 5 weeks, auditory-sensorimotor EEG co-activation was observed already after the first session of 20 min. This effect increased after the training was complete. Moreover, piano training was associated with sensory hand area activation during listening. The co-activation of the auditory-sensorimotor network regions while listening to learned musical stimuli has been identified in the previously discussed studies investigating musical training with fMRI (Amad et al., 2016; Li et al., 2018, 2019; Wollman et al., 2018).

#### Predispositions

Predispositions are factors which are positively associated with the rate at which a new skill is acquired or the skill level attained at the end of the training. Thus, they can strongly influence the time needed to achieve musical expertise or the potentially achievable expertise level, resulting in individual differences in musical skill acquisition. So far, very few studies examined how various predisposing factors affect the outcomes of the learning process in a short time-scale.

Two recent behavioral studies found attitude and intelligence might be indicative of learning success (Bianco et al., 2019; Burgoyne et al., 2019). In the first study (Bianco et al., 2019), as participants were learning to play melodies on a keyboard, the accuracy and asynchrony of keystrokes were taken as measures of motor performance. The better liked melodies were also learned more easily, showing less asynchrony in the learning trials. Moreover, participant’s motivation, reflected in the individual’s task-perceived competence, correlated with the performance on the not-liked melodies. These outcomes are in line with previous findings on the role of reward and pleasure in learning music performance (Schultz, 2000). Namely, participant’s positive attitude toward a certain piece of music before training positively influenced the ability to learn it and achieved performance proficiency. In the second study (Burgoyne et al., 2019), participants underwent a short session of learning how to play Happy Birthday on an electronic piano. General intelligence was found to account for 21.4% of the variance in skill acquisition, while the effects of mindset and aptitude were negligible and non-significant. This result points to the potential role which intelligence plays in the facilitation of early learning.

The topic of predicting participants’ performance after training was a subject of interest in two of the aforementioned longitudinal fMRI studies. In the research of Herholz et al. (2016), distinct neural structures were identified, where stronger recruitment during listening to a known melody before training predicted the slope of participant’s learning curve. The activation of these structures, including the right Heschl’s gyrus, left mid-premotor cortex, bilateral caudate nucleus, and right hippocampus, did not change in response to training; therefore, it can be understood as a specific predictor of learning success. In the context of musical training and auditory cognition, the activation of Heschl’s gyrus and hippocampus can be attributed to the encoding of stimuli, while the activation of premotor cortex and caudate nuclei to aspects of motor control. Heschl’s gyrus is a region commonly associated with auditory pitch processing and discrimination (Patterson et al., 2002), and has been previously identified as a predictor of auditory learning success (Wong et al., 2008; Zatorre et al., 2012). In the cello training study (Wollman et al., 2018), fMRI and DTI were used to identify neural substrates which could predict training success. The activation of the pre-supplementary motor area during passive listening of trained melodies and its functional connectivity with the auditory cortex before training were predictive of subsequent training success after four weeks.

Additionally, in a DTI study, microstructural organization of the right arcuate fasciculus (i.e., volume and fractional anisotropy) before training predicted the learning rate and learning speed in rhythm- and melody-learning tasks (Vaquero et al., 2018). Higher fractional anisotropy in the arcuate fasciculus was associated with musical training in previous studies (Halwani et al., 2011). Reduced volume of the arcuate fasciculus may be associated with congenital amusia (Loui et al., 2009) and predicts non-recovery from acquired amusia after a stroke (Sihvonen et al., 2017).

### Limitations of Longitudinal Studies and Suggestions for Future Research

The majority of longitudinal studies in musical training employed very short training periods (a matter of minutes or hours within a single neuroimaging session) (Chen et al., 2012; Brown and Penhune, 2018; Vaquero et al., 2018; Bianco et al., 2019; Burgoyne et al., 2019; Tavor et al., 2020). Such short learning periods can inform us only on acute changes in brain function and organization while learning a new skill. This set-up does not include a control group or a control paradigm. Therefore, the observed effects might represent a mixture of learning-induced plasticity and a repetition/task-familiarity effect. The longitudinal studies which investigate long-term learning over the periods of weeks or months are fewer, as these studies pose a much greater organizational and financial challenges. While looking at long-term changes, it is crucial to introduce an adequate control condition/group (Makin and Orban de Xivry, 2019). For structural neuroplasticity or changes in functional connectivity, a matched passive control group that does not undergo training may be sufficient. Having a matched passive control group that is followed up as frequently as a training group allows to distinguish between training-unrelated time effects and effect of task repetition from learning-induced plasticity. A different approach is within-subject control, whereby before training commences, participants undergo a control timepoint scan (e.g., Herholz et al., 2016). This allows to assess the magnitude of functional/structural changes in a given period in the same group of individuals before they start learning a new skill. Having a reliable control group may be particularly difficult if one is interested in task-related plasticity changes. Naive individuals will not be able to perform the same task as trained individuals (e.g., play an instrument). In this case, a carefully designed control condition may be introduced (e.g., Wollman et al., 2018). These are crucial methodological issues which should be carefully considered by those who plan a longitudinal study. For additional information on structural and functional neuroplasticity methods, their strengths and weaknesses, see Table 1.

## Summary and Future Directions

Neuroplasticity is a process of structural and functional brain adaptation to achieve new skills in response to internal and external stimuli. Structural changes in the gray and white matter and functional changes of the brain activation patterns are intertwined and can develop in parallel. Importantly, they happen over time and in response to a specific new skill.

Cross-sectional approach, that is a comparison of individuals with varying levels of a given skill, is particularly useful in musical training research as mastering a technique of playing an instrument usually takes years of dedication and practice. It has been estimated that professional and exceptional musicians accumulate over 10,000 h (approximately 10 years’ worth of training) of individual practice by the time they are 20 years old (Ericsson et al., 1993). Longitudinal study of such duration would be extremely costly and difficult to perform. Additionally, correlations between structural volumes and the number of years of practice indicate that structural variability is a result of experience-dependent plasticity rather than merely initial individual differences. However, cross-sectional studies, as any research comparing independent groups of individuals, is confounded by developmental differences in specific group experiences (Poldrack, 2000).

Extensive research has been performed using neuroimaging and musical training, providing evidence in favor of training-related plasticity. Cross-sectional studies identified structural and functional differences between the brains of musicians and non-musicians in regions related to motor control and auditory processing, such as the Heschl’s gyrus, the primary motor and premotor areas, parietal areas, and the fiber tracts connecting them. The auditory-premotor-parietal network seems to be engaged in musical training and changed by it, as reflected in the longitudinal studies. Functional changes during passive listening and music production in the motor network and its connectivity with the auditory system were associated with musical training of naive participants. Parallel changes within the motor system and between the motor and auditory systems were revealed for structural connectivity. These findings are further corroborated by a recent study showing rapid changes in mean diffusivity of the premotor region, which reflects microstructural alterations (Tavor et al., 2020). Functional, microstructural and functional connectivity changes in the cerebellum, a known structure involved in motor coordination and motor learning, were observed in association with musical training.

To shed light on the nature-or-nurture question, one might compare the consistency in the outcomes of cross-sectional and longitudinal studies. The differences between musicians and non-musicians identified in cross-sectional studies, which overlap with changes found in longitudinal studies, may be considered to be associated, at least partly, with musical training itself. Indeed, results from both experimental approaches often point to the same brain structures being associated with musical training (e.g., the auditory, parietal and the premotor cortices, belonging to the dorsal auditory stream), although using different neuroimaging methods [structural or functional (Table 1)].

Many cross-sectional studies report differences between musicians and non-musicians at the structural level, which have been studied in a couple of longitudinal experiments. For example, microstructural differences (measured as a reduction in mean diffusivity) associated with musical training in the cerebellum, responsible for motor coordination (Tavor et al., 2020), were in line with cross-sectional research (Schmithorst and Wilke, 2002; Abdul-Kareem et al., 2011), providing evidence in favor of musical-training related neuroplasticity. Another longitudinal study, which focused on predispositions and used structural measures showed that the fractional anisotropy and the tract volume of the arcuate fasciculus were associated with faster learning rate in a musical task (Vaquero et al., 2018). This outcome is in agreement with cross-sectional studies on musicians and amusics (Loui et al., 2009; Halwani et al., 2011; Sihvonen et al., 2017; de Manzano and Ullén, 2018), where larger tract volumes and higher fractional anisotropy of the arcuate fasciculus were associated with musical training (Halwani et al., 2011; de Manzano and Ullén, 2018), and smaller tract volume and fractional anisotropy were associated with amusia disorder (Sihvonen et al., 2017). Those results suggest that the size and the microstructure of the arcuate fasciculus might be a predisposition, i.e., affect individual musical skill acquisition.

The presented here longitudinal studies concentrated mostly on the functional aspects of brain plasticity (task fMRI, functional connectivity, EEG, MEG) (Table 2). Listening to music was often employed in both cross-sectional and longitudinal experiments, as a valid task at all musical skill levels. In cross-sectional studies, during passive music listening, greater activation of brain structures related to music processing and production in the temporal and premotor cortices, respectively, was found in musicians compared to non-musicians (Ohnishi, 2001; Seung et al., 2005; Bangert et al., 2006; Limb et al., 2006; Habermeyer et al., 2009; Herholz et al., 2011; Kuchenbuch et al., 2014; Bianchi et al., 2017). Similarly, in longitudinal studies, greater activation of these structures while listening to music was found after training (Lappe et al., 2008; Pantev et al., 2009; Paraskevopoulos et al., 2012; Herholz et al., 2016; Brown and Penhune, 2018; Wollman et al., 2018). Additionally, parallel findings of increased activation in various areas of the parietal lobe during passive music listening tasks have been found in cross-sectional studies comparing musicians to non-musicians (Seung et al., 2005; Limb et al., 2006; Oechslin et al., 2013) and in longitudinal studies after musical training (Herholz et al., 2016; Wollman et al., 2018).

**TABLE 2:** Summary of findings of longitudinal neuroimaging studies on musical training and behavioral studies on predispositions to learning how to play an instrument.

Study	Design							Outcomes	
Author Year	*N* =	Age	Control	Instrument	Measurement	Number of time points (TP)/length of training	Experimental design/task	Post > Pre comparison	Prediction of learning success
**MRI**									
Chen et al., 2012	16	Range: 20–34, M = 27.13	resting silence	keyboard	task fMRI	single fMRI session	play the piano	↓ activation left dorsal and ventral PMC ↑ activation right retrosplenial cortex, orbitofrontal cortex, frontal pole	↓ activation left dorsal PMC while training
							listen to trained melodies	↓ activation bilateral STG	
Brown and Penhune, 2018	16	M = 24.2	listening, resting silence	keyboard	task fMRI	single fMRI session	play the piano	↓ activation parietal regions compared to listening ↓ activation superior parietal, inferior parietal, auditory cortex, PMC ↑ occipital cortex	
							listen to trained melodies	↑ occipital, temporal cortex, PMC, cerebellum ↓ auditory cortex	
Tavor et al., 2020	32	M = 25.7, SD = 3.1	pre-post design	piano	mean diffusivity	TP0 (before training) TP1 (TP0 + 45 min)	–	↓ mean diffusivity left PMC, bilateral superior cerebellum, left MTG	
						TP2 (TP1 + 40 min)	–	↓ mean diffusivity left PMC, bilateral superior cerebellum, left MTG, lingual gyrus	
Amad et al., 2016	15	16–19	control group N = 16	drums	rs-fMRI	TP0 (before training) TP1 (TP0 + 8 weeks)	–	↑ FC between posterior bilateral STG (seed) and whole brain ↑ FC between STG (seed) and premotor region and right parietal lobe ↓ FC between STG (seed) and cerebellum	
Herholz et al., 2016	15	Range: 20–34, M = 25.6	control timepoint before training	piano	task fMRI	TP0 (before training) TP1 (TP0 + 6 weeks)	listen (familiar vs random)	↑ activation left dorsal PMC	↑ activation right Heschl’s gyrus, right hippocampus ↓ activation medial frontal areas and frontal pole
							listen & imagine (trained vs untrained)	↑ activation PMC, dorsolateral prefrontal cortex, bilateral posterior parietal cortex, bilateral cerebellum	–
							imagine (trained vs untrained)	↓ activation lateral occipital cortex	–
Wollman et al., 2018	13	Range: 20–31, M ± 26 ± 4	listen to untrained melody	cello	task fMRI	TP0 (before training) TP2 (TP0 + 4 weeks)	listen	↑ activation pre-SMA & SMA ↑ activation right dorsal PMC ↑ activation left posterior parietal cortex	↑ activation right STG, right putamen, left middle frontal gyrus, pre-SMA
					task fMRI	TP1 (TP0 + 1 week) TP2 (TP1 + 3 weeks)	play the cello	–	↑ activation right STG, hippocampus
					task fMRI connectivity	TP0 (before training) TP2 (TP0 + 4 weeks)	listen	↑ FC between superior parietal lobe (seed) and bilateral auditory cortex ↑ FC between superior parietal lobe (seed) and SMA	↑ FC between pre-SMA (seed) and bilateral superior temporal gyrus
					rs-fMRI	TP0 (before training) TP2 (TP0 + 4 weeks)	rest pre-training	–	↑ FC between pre-SMA (seed) and planum temporale
Li et al., 2018	29	exp. group: M = 23.10 ± 1.37 control group: M = 23.33 ± 1.39	control group *N* = 27	piano	rs-fMRI	TP0 (before training) TP1 (TP0 + 24 weeks)	–	↑ FC between the right postcentral and the right precentral gyri ↑ FC between the sensorimotor and the auditory region	–
					DTI	TP0 (before training) TP1 (TP0 + 24 weeks)	–	↑ FA between the sensorimotor and the auditory regions	–
Li et al., 2019	29	exp. group: M = 23.10 ±1.37 control group: M = 23.33 ±1.39	control group *N* = 27	piano	rs-fMRI	TP0 (before training) TP1 (TP0 + 24 weeks)	–	↑ flexibility in the visual and auditory cortices	–
Vaquero et al., 2018	35	M = 22.16, SD = 2.56		piano	DTI	TP0 (before training) TP1 (TP0 + 15*min)	melody training	–	↑ volume right anterior arcuate fasciculus
	24			piano	DTI	TP0 (before training) TP1 (TP0 + 10*min)	rhythm training	–	↑ volume right arcuate fasciculus (long segment) ↑ FA right arcuate fasciculus
**EEG/MEG**									
Lappe et al., 2008; Pantev et al., 2009	10	Range: 24–38	control group *N* = 10 (auditory training)	piano	MEG	TP0 (before training) TP1 (TP0 + 2 weeks)	sensorimotor-auditory vs auditory training	↑ amplitude MMN auditory cortex	–
Paraskevopoulos et al., 2012	12	M = 25.86, SD = 3.17	Control group *N* = 12 (auditory-visual training)	Piano	MEG	TP0 (before training) TP1 (TP0 + 2 weeks)	auditory-visual-sensorimotor vs auditory-visual training - audio-visual task	↑ activation MMN STG (Broca area 22)	–
Bangert and Altenmüller, 2003	9	M = 26.2 ±5.3	control group *N* = 8 (random pitch assignment)	piano	DC-EEG	TP0 (before training) TP1 (TP0 + 20 min)	conventional vs randomly changing pitch assignment	↑ activation left primary sensorimotor cortex ↑ activation right fronto-temporal electrodes	–
						TP0 (before training) TP2 (TP0 + 5 weeks)	conventional vs randomly changing pitch assignment	↑ activation right fronto-temporal electrodes	–
**Behavioral**									
Bianco et al., 2019	27	M = 24.74 ±5.03		piano	n/a	TP0 (before training) TP1 (TP0 + 12*min)	–	–	↑ liking attitude toward learned melody
Burgoyne et al., 2019	161	Range: 18–25 M = 20.19 SD = 1.47		piano	n/a	TP0 (before training) TP1 (TP0 + 6*min) TP2 (TP1 + 6*min)	–	–	↑ intelligence

The studies had to incorporate at least two measurement time points (before and after musical training). M, mean; SD, standard deviation; PMC, premotor cortex; STG, superior temporal gyrus; SMA, supplementary motor area; pre-SMA, pre-supplementary motor area; MTG, middle temporal gyrus, FC, functional connectivity; FA, fractional anisotropy; *, training duration estimated from manuscript text.

Altogether, these outcomes provide evidence in favor of musical training-related functional neuroplasticity in the dorsal auditory stream but also point to the existence of predispositions which might affect individual musical skill acquisition.

The engagement of the auditory-premotor-parietal network of the dorsal auditory stream was common despite that presented studies were performed using various instruments (piano, keyboard, cello, drums) and can be also observed in singing (Segado et al., 2018). This provides grounds to anticipate that any training that requires music production would change at least some aspects of this network. Although those structures consistently appear in both cross-sectional and longitudinal experiments, the size and sometimes the direction of the effect can vary greatly from study to study. Those differences might stem from the differences in study design (different training paradigms, timescales, measurement methods) or reflect different aspects of the neuroplasticity process. After all, we do not expect the training-related changes to be linear, nor concurrent within the whole brain (Blumenfeld-Katzir et al., 2011; Sagi et al., 2012).

At the moment, the dynamics of the network adaptations caused by musical training are still under investigation and many questions remain unanswered. Does a pattern exist where various brain structures respond one after another, or do they change in parallel? How are the structural and functional changes related in time? As most of the neuroplastic changes are expected early in the training, to answer these, and other, questions in the future, it is tempting to limit the training duration to its initial period. Long-lasting research is costly and training duration should be carefully considered. Yet, while valid, the short-term approach might not be sufficient to capture the full scope of the changes. In its INITIAL period, we expect plasticity processes to be complex, dynamic, and non-linear, therefore, it is desirable to perform multimodal longitudinal studies with multiple measurements (time points) spread over the course of training. For example, certain dynamic changes in plasticity can be detected within minutes of training by measuring diffusivity (Tavor et al., 2020) or changes in brain activation (fMRI) (Chen et al., 2012; Brown and Penhune, 2018). Further changes in functional brain connectivity are present following longer training periods (Amad et al., 2016; Wollman et al., 2018); however, there is a scarcity of studies employing a multimodal approach to neuroplasticity repeatedly over extended intervals. An exception here is a recent study in which functional and structural brain changes in sighted tactile Braille learners were investigated multiple times over an 8-month period (Matuszewski et al., 2021). Employing a multi-contrast MRI approach allowed to describe and contrast functional and structural neuroplastic mechanisms underlying complex cognitive and sensorimotor learning, at a macroscopic level over an extended period.

Notably, the longitudinal studies presented above used a wide variety of training paradigms and durations, instruments and tools. In some, participants practiced for 30 min, five times per weeks, for six weeks [(Herholz et al., 2016); piano, fMRI]; in others, for 60–90 min, two times per week, for two weeks [(Wollman et al., 2018); cello, fMRI and functional connectivity] or 30 min, three times per week, for 8 weeks [(Amad et al., 2016); drums, functional connectivity]. Given this variability, it is striking that the outcomes generally include similar areas of the auditory-motor-parietal network, but not surprisingly, they differ in fine details. Importantly, the areas of the dorsal auditory stream are reported to be associated with musical training also in cross-sectional studies, when comparing musicians to non-musicians.

Fortunately, cross-sectional studies provide us with clues of what effects could be associated with lifelong training. Based on available evidence, the differences in brain function and morphology between musicians and non-musicians can be attributed to both predispositions and effects of musical training. Therefore, “the musical brain” is likely a product of both the natural human neurodiversity and the training practice, in varying, and yet largely unknown, proportions (Barrett et al., 2013).

## Author Contributions

AO and AM: conceptualization. AO and MG: literature search and selection, and writing of the original draft of the manuscript. AM: funding acquisition. All authors contributed to the drafting and revising of the article and approved the submitted version.

## Conflict of Interest

The authors declare that the research was conducted in the absence of any commercial or financial relationships that could be construed as a potential conflict of interest.
